# Sperm capacitation in bulls, rams, bucks, and boars: molecular mechanisms and species-specific differences

**DOI:** 10.3389/fvets.2026.1814057

**Published:** 2026-04-13

**Authors:** Abdulkadir Kaya, Raheem Murray, Mustafa Bodu, Mustafa Hitit, Erdogan Memili

**Affiliations:** 1 Department of Reproduction and Artificial Insemination, Faculty of Veterinary Medicine, Kırıkkale University, Kırıkkale, Türkiye; 2 College of Agriculture, Food, and Natural Resources, Prairie View University, Prairie View, TX, United States; 3 Department of Reproduction and Artificial Insemination, Faculty of Veterinary Medicine, Selcuk University, Konya, Türkiye

**Keywords:** bull, capacitation, comparative reproduction, goat, livestock, signal transduction, sperm

## Abstract

Capacitation is the process by which sperm undergoes physical, biochemical, and molecular changes to acquire the ability to fertilize an oocyte. The first stage occurs after ejaculation with the removal of cholesterol from the cell membrane, reorganization of the cell membrane, and permission of ion entry into the cell. Intracellular alkalization leads to signaling, resulting in the basic capacitation cascade (sAC-cAMP-PKA-PTP). Consequently, the process of hyperactivation and preparation for the acrosome reaction is completed. Significant interspecies differences are observed in farm animals, and identifying these differences will make important contributions to the development of reproductive technologies. Bull sperm is mostly dependent on heparin-like glycosaminoglycans; however, in ram, seminal plasma proteins are shown to play important roles during capacitation. In goat, the rapid and stress-sensitive capacitation mechanism and the tight zinc-dependent *in vivo* control of boar sperm are important. Furthermore, early and premature capacitation caused by cryopreservation is called cryocapacitation and directly affects fertilization success. Species-specific capacitation mechanisms have been investigated; however, understanding differences will be beneficial using *omics* approaches in fertilization prediction and in developing cryopreservation strategies. Moreover, this review compares the molecular mechanisms of sperm capacitation in bulls, rams, bucks, and boars. It highlights species-specific differences and their impact on reproductive technologies.

## 1 Introduction

**Figure 1:**
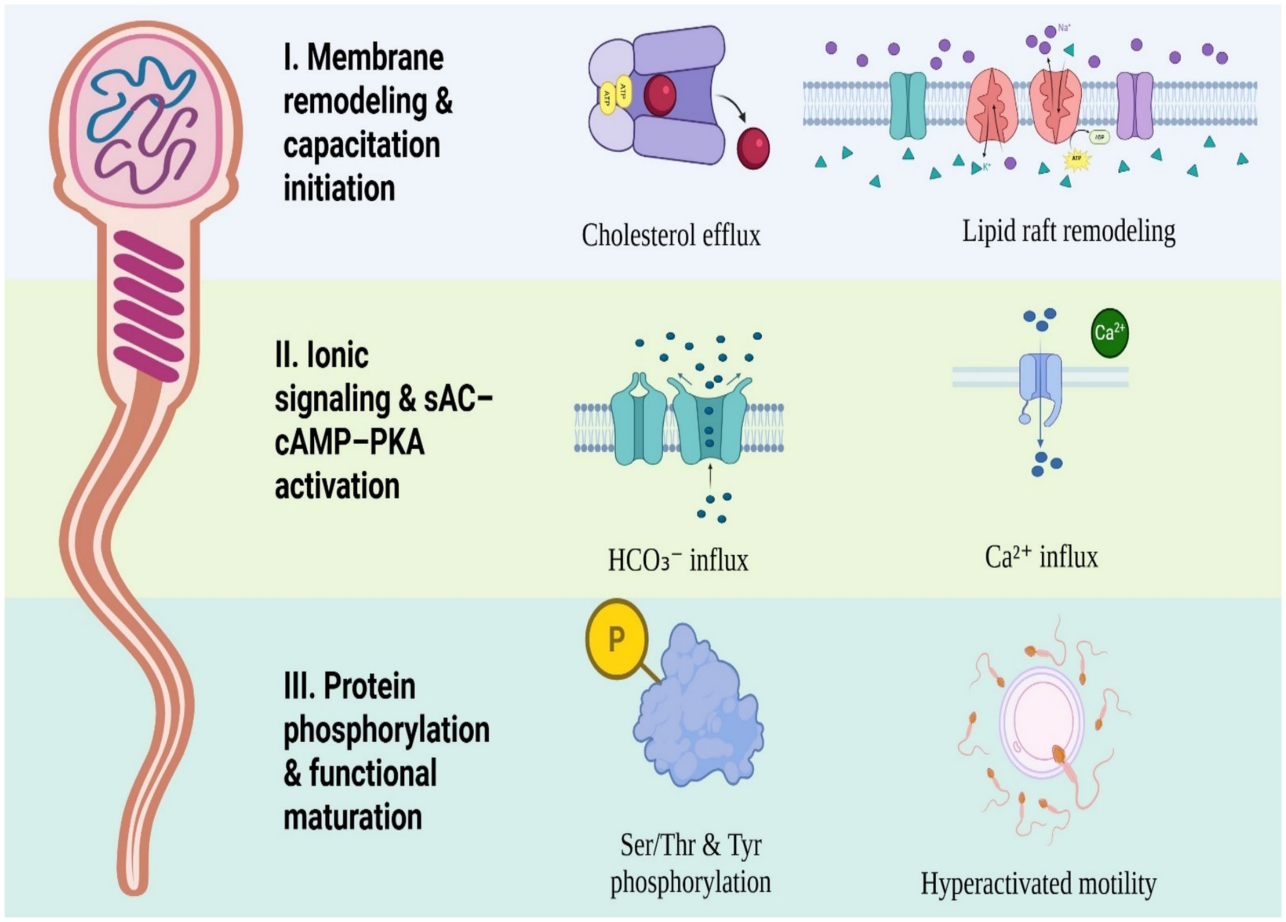
Schematic overview of the capacitation cascade in mammalian sperm highlighting membrane, ionic, and phosphorylation-dependent events (created with BioRender.com).

Sperm capacitation refers to the process in which sperm undergo physical, biochemical, and molecular transformations after ejaculation, then acquire the ability to fertilize. The cascade of capacitation consists of consecutive events; the removal of cholesterol from the plasma membrane, increased membrane fluidity, influx of bicarbonate and calcium, activation of soluble adenylyl cyclase (sAC), cAMP-dependent protein kinase A (PKA) signaling, and final protein tyrosine phosphorylation (PTP) (1, 2) (Table 1). Consequently, sperm gains the capacity to bind to the zona pellucida, participate in the acrosome reaction, penetrate the oocyte via hyperactivation, and finally achieve gamete fusion (3). Recently, some methodologies like, flow cytometry (4), time-lapse epifluorescence imaging of zinc ion dynamics (5), and morphometric analyses based on combined fluorescence-electron microscopy (6) have strengthened our understanding of the molecular and structural events underlying the capacitation cascade. Despite numerous studies on sperm capacitation, key differences between species remain incompletely understood, particularly among livestock species with high economic value. Differences in the sperm membrane, signaling pathways, and sensitivity to capacitating factors suggest that capacitation is regulated in a species-specific manner. A comparative understanding of these differences is essential for improving assisted reproductive technologies and cryopreservation strategies. Therefore, the objective of this review is to compare the regulation and timing of sperm capacitation in bull (*Bos taurus*), ram (*Ovis aries*), buck (*Capra hircus*), and boar (*Sus domesticus*). These species represent economically important farm animals and differ in their physiological and molecular characteristics during sperm capacitation (Figure 1).

**Table 1:** Key molecular events involved in sperm capacitation.

Molecular event	Main pathway/molecule(s)	Functional outcome	References
Cholesterol efflux	Albumin, HDL, heparin-like glycosaminoglycans; BSP/PDC-109 proteins	Increased membrane fluidity; redistribution of lipid rafts; initiation of capacitation	(14, 94)
Bicarbonate influx	HCO₃^−^ → soluble adenylyl cyclase (sAC)	Rapid increase in intracellular cAMP; activation of downstream signaling	(14)
Physiological ROS generation	H₂O₂, NO; mitochondrial and membrane-associated oxidases	Redox-dependent signaling supporting capacitation without oxidative damage	(57, 95)
cAMP production and PKA activation	cAMP–PKA–AKAP signaling complexes	Phosphorylation cascades coordinating motility and capacitation	(12, 96)
Protein tyrosine phosphorylation	PKA-dependent activation of tyrosine kinases	Acquisition of fertilizing ability; preparation for hyperactivation and acrosome reaction	(94, 96)
Ca^2+^ influx	CatSper channels; membrane depolarization	Induction of hyperactivated motility; regulation of flagellar beating	(16)
Actin cytoskeleton remodeling	F-actin polymerization; actin-binding proteins	Stabilization of sperm head; prevention of premature acrosome reaction	(29, 38)
Displacement of seminal plasma–derived inhibitory proteins	BSP/PDC-109, RSVP14/20, SPINK3, SERPINE2; albumin-mediated dissociation and sperm surface protein reorganization	Relief of inhibitory constraints on cAMP/PKA-dependent signaling, allowing full establishment of the capacitated state permissive for zona pellucida binding and acrosome reaction	(8, 44, 53)

## 2 Mechanism of capacitation cascade

### 2.1 Early stage of capacitation

Early stage of capacitation is characterized by the removal of cholesterol from the plasma membrane and the rearrangement of membrane structure through lipid raft reorganization in the female reproductive system by the influence of albumin and high-density lipoproteins (7–9). As a result, cholesterol depletion induces the redistribution and aggregation of lipid rafts, which are dynamic micro-spaces rich in cholesterol and sphingolipids that serve as platforms for signaling molecules (10). Sphingolipids and glycolipids in the cell membrane play a critical role in lipid raft organization associated with hyperactivation and acrosome exocytosis (11). Thus, a more fluid cell membrane allows calcium and bicarbonate ions to enter the cell, enabling sAC activation, increased intracellular cAMP, and PKA activation (7, 9).

### 2.2 Ion flux and intracellular signaling phase

The generation of the capacitation signal is dependent on an increase in intracellular bicarbonate (HCO₃^−^) and calcium (Ca^2+^) (12–14). Following ejaculation, sperm cells entering the female reproductive system are exposed to high bicarbonate concentrations originating from both seminal plasma and the oviduct environment, which then initiates the signaling pathways necessary for capacitation (2, 15). Increased membrane fluidity facilitates bicarbonate entry into the sperm cell, leading to an elevation of intracellular pH and initiation of capacitation. This bicarbonate influx is mediated by Na^+^/HCO₃^−^ transporters and Cl^−^/HCO₃^−^ exchangers (1, 13, 15). On the other hand, the Cystic Fibrosis Transmembrane Conductivity Regulator (CFTR) plays a role in regulating signaling, and carbonic anhydrase (CA) catalyzes the hydration of CO₂ to bicarbonate (2, 14, 15). Alkalization is a prerequisite for the activation of CatSper channels (16), a pH-sensitive, voltage-gated ion channel located in the flagellum plays a role in calcium influx into the cell (17, 18). The CatSper channel also senses signals from progesterone, prostaglandins, and zona pellucida proteins, and links extracellular stimuli from the female reproductive system to intracellular sperm Ca^2+^ dynamics (16, 18, 19). Therefore, the uptake of bicarbonate and calcium into the cell is an important factor in maintaining capacitation. Dilution and cryopreservation, which are known to affect the sperm cell membrane, have been reported to disrupt the mechanism of capacitation and induce premature activation (20).

Ion influx and alkalization hyperpolarize the cell membrane, stabilizing membrane potential and structure via CFTR-Epithelial Na^+^ Channel (ENaC) interaction, leading to sAC activation (2). Mechanistically, calcium (Ca^2+^) influx works synergistically with HCO₃^−^ to stabilize ATP binding on the sAC and increase cAMP synthesis (21–23). Furthermore, calcium is a key ion in the process from the initiation of capacitation to zona pellucida penetration and gamete fusion (19, 23, 24). Consequently, the increase in both ions leads to the conversion of ATP to cAMP and inorganic pyrophosphate (PPi) (13, 21, 22). It has been shown that the capacitation cascade can be stimulated by exogenous cAMP analogs, demonstrating the key role of cAMP (25, 26). Intracellular cAMP accumulation is a crucial step in sperm capacitation and leads to the activation of two main downstream effectors in which (1) cAMP-activated exchange protein (EPAC) regulates processes such as Ca^2+^ signaling and acrosome reaction and (2) protein kinase A (PKA), whose importance we will emphasize in the next section, activates protein phosphorylation, which underlies hyperactivation and acrosome reaction (13, 22, 27).

### 2.3 Final stage of capacitation

Protein phosphorylation is a key regulatory mechanism during sperm capacitation, and this process is characterized by a progressive increase in protein tyrosine phosphorylation. Most tyrosine-phosphorylated proteins are localized in the fibrous sheath of the sperm flagellum, where they regulate hyperactivated motility and support energy metabolism (28, 29). In addition to the flagellum, tyrosine phosphorylation is also observed in the sperm head, particularly in the acrosomal and equatorial regions, where it is associated with zona pellucida recognition and preparation for acrosomal exocytosis (30). In this way, phosphorylation plays an integral role in the coordination of motility, metabolism, and fertilization (31, 32).

PKA, via cAMP, is a key regulatory element at the functional remodeling in sperm through ionic signaling. PKA alters both the structural properties and functional activities of target proteins by adding a phosphate group (–PO₄) to serine (Ser) and threonine (Thr) residues, and serine/threonine phosphorylation is one of the early and prerequisite molecular events that occur during capacitation (12, 14, 24). At the same time, motor proteins such as dynein and tubulin, as well as various signaling proteins, are affected by this phosphorylation, undergoing reorganization in sperm motility and energy utilization (33).

On the other hand, PKA is to initiate a second wave of phosphorylation in the capacitation process by activating tyrosine kinases such as Src family kinases and Pyk2. Subsequently, transition allows for the extension of serine/threonine-based signaling through tyrosine phosphorylation. During this stage, protein phosphatase-1 (PP1) is inhibited via CaMKII, thus maintaining the phosphorylation status of downstream proteins. PP1 inhibition acts as a critical mechanism for ensuring signal continuity during capacitation. The ionic signaling of PKA is spatially organized by *α*-kinase binding proteins (AKAPs), which enable localized and controlled phosphorylation by fixing PKA to specific subcellular regions (32, 34). While kinase plays a role in regulating sperm energy metabolism through phosphorylation, the AKAP4 protein binds PKA to the flagellum, allowing the signaling pathway to exert flagellum-specific effects (35, 36).

In addition, the activation of ERK1/2 and p38 proteins is initiated via the mitogen-activated protein kinase (MAPK) pathway. PKA activation increases calcium influx into the cell by contributing to the phosphorylation of specific Ca^2+^ channels along with their associated regulatory proteins. Increased Ca^2+^ levels activate kinases such as CaMKII and PKC, which, in coordination with the chaperone protein Hsp90, thereafter facilitate the activation of the MAPK signaling pathway. Meanwhile, activation of the MAPK cascade leads to increased sperm flagellum motility while simultaneously achieving the cellular preparatory process necessary for the acrosome reaction (37).

Focal adhesion kinase (FAK) in the cytoskeleton, is activated in this process, then stimulating ERK2. FAK-mediated signaling enables the conversion of cellular mechanical stimuli into biochemical responses. ERK2 phosphorylates GEF-H1, subsequently triggering the activation of small GTP-binding proteins. Rho GTPases play a central role in regulating cytoskeletal dynamics of sperm cell, and activation of RhoA and Rac1 activates LIM kinase and phosphorylates cofilin (24, 29, 38). Consequently, it suppresses the degradation of actin filaments, promotes polymerization, and ensures the formation of a stable actin scaffold necessary for acrosomal exocytosis (38, 39).

Finally, PTP supports cytoskeletal remodeling and actin polymerization, then the resulting hyperactivation facilitates sperm-egg interaction through protein regulation in the cell membrane and prepares sperm for the acrosome reaction (12, 40, 41).

## 3 Comparative molecular analysis of species-specific differences

### 3.1 Bull (cattle)

Bull sperm exhibits a distinct capacitation response to glycosaminoglycans (GAGs), particularly heparin and structurally analogous molecules, which are widely used as physiological inducers of capacitation (42, 43). Nevertheless, capacitation in bull sperm is not exclusively dependent on GAGs, and additional factors such as albumin, bicarbonate, calcium, and seminal plasma proteins (e.g., BSP proteins) also contribute to the regulation of this process (1, 44, 45).

After ejaculation, heparin starts functioning at the sperm plasma membrane, especially in bull sperm, where it is often used to help sperm become more active. Heparin first attaches to a certain group of seminal plasma proteins that are all called “binder of sperm” (BSP) proteins. PDC-109 (BSP-A1/A2), BSP-A3 (PDC-86), BSP-30 kDa (BSP-30A/B), and BSP5 (BSP-A5) are all members of this protein family (44–46). Consequently, PDC-109 extensively covers the sperm surface, which helps cholesterol and phospholipids leave the plasma membrane (44, 45). Lipid efflux greatly increases the fluidity and disorder of membranes. This process prepares bull spermatozoa for downstream signaling related to capacitation, rather than being a mechanism that is shared by all mammals (42).

Phosphorylation signaling events during capacitation are well-defined, with heparin-induced capacitation acting as a reliable and species-specific experimental model. Chamberland et al. (42) were the first to find a sperm protein that was about 50 kDa and only underwent intense phosphorylation during heparin-induced capacitation. This was a signaling event that had not been seen in other species. Later studies showed that this protein was made up of partial proteolytic fragments of AKAP4, which is a key structural part of the flagellar fibrous sheath and an important scaffold for PKA-mediated signaling in sperm (31, 32). Mostek-Majewska et al. (47) also found more PKA-phosphorylated targets that are only found in bull sperm capacitation. They also saw that tyrosine phosphorylation of PRKAR1A, the regulatory subunit of PKA, supports the idea that there is a bull-specific autoregulatory loop that keeps PKA signaling going during capacitation (47).

In accordance with these capacitation-specific signatures, numerous proteins have established connections to bull fertility that intersect with the capacitation-associated proteome. Protamine-1 (PRM1) is essential for chromatin condensation and DNA integrity, with increased levels associated with diminished DNA fragmentation and enhanced fertility outcomes (48, 49). HSP-70 aids in cryo-tolerance, mitochondrial stability, and membrane integrity, functions that are especially important for processing semen. Additionally, AKAP4, in addition to its signaling role during capacitation, has recently been confirmed as a dependable marker of sperm motility and fertilization potential (50). We also found that bulls with high fertility have higher levels of structural and oocyte-activating proteins like ODF2 and PAWP, which help with hyperactivation, the acrosome reaction, and the fusion of sperm and oocytes (51).

### 3.2 Ram (sheep)

Ram sperm, particularly when compared to the bull, whose capacitation mechanism is significantly dependent on GAGs, rely on its proteomic structure, where immune-protective seminal plasma proteins are critical for capacitation (42, 43). Ram sperm are also more dependent on seminal plasma factors that stabilize membranes, act as decapacitating agents, and modulate immune responses, thereby preventing premature capacitation along the female reproductive tract (vagina, cervix, and uterus) and ensuring that capacitation is completed at the appropriate site within the oviduct (52, 53).

RSVP14 and RSVP20 are seminal plasma proteins identified in ram semen that act as decapacitating factors. These proteins bind to the sperm surface and help maintain a non-capacitated state by preventing the increase in protein tyrosine phosphorylation during capacitation (53). RSVP20, on the other hand, maintains sperm membrane integrity by binding to heparin-like molecules and plays a dual role in both inhibiting and fine-tuning the process by increasing sperm-zona pellucida binding (52–54). Similarly, members of the BSP family show distinct functional effects on sperm physiology. BSP1 is known to interact with sperm membranes and participate in lipid remodeling processes associated with capacitation, whereas BSP5 has been reported to modulate sperm viability and capacitation dynamics under certain conditions (46). Another protein involved in preventing premature capacitation is serine protease inhibitor Kazal-type 3 (SPINK3), a seminal plasma protein that binds to the sperm surface and stabilizes the plasma membrane, thereby helping maintain sperm in a non-capacitated state and modulating calcium influx (55).

Seminal plasma proteins play important roles in stabilizing the sperm membrane, regulating ion channels, and controlling oxidative and apoptotic processes (52, 56). These proteins also help maintain sperm viability during storage and transport by regulating signaling pathways involved in capacitation (52, 56). Signaling pathways such as c-Jun N-terminal kinase (JNK) and p38 mitogen-activated protein kinase (p38 MAPK) regulate cellular stress and apoptosis, and their excessive activation may negatively affect sperm capacitation and survival (56).

Despite having a well-developed antioxidant defense system, ram sperm are highly susceptible to oxidative damage (57). However, controlled production of reactive oxygen species (ROS) is essential for sperm capacitation and contributes to membrane remodeling and protein tyrosine phosphorylation (57, 58). However, excessive ROS production can lead to lipid peroxidation, protein oxidation, DNA fragmentation, and premature capacitation or the acrosome reaction (59, 60). The high sensitivity of ram sperm to ROS is largely related to the polyunsaturated fatty acid (PUFA)-rich composition of the plasma membrane and their limited antioxidant capacity, which mainly depends on enzymes such as superoxide dismutase (SOD), glutathione peroxidase (GPX), and peroxiredoxins (PRDXs) (61).

### 3.3 Buck (goat)

Capacitation in buck sperm occurs faster than in many other livestock species. This feature influences fertilization efficiency and makes buck sperm particularly sensitive to environmental and biochemical conditions during semen handling and storage. Under *in vitro* conditions, functional changes associated with capacitation can be detected rapidly (62, 63), and these changes may appear within approximately one hour after induction (64). When compared with other species such as bull and ram, the duration of capacitation in bucks is generally shorter (62, 64). Although this rapid response can facilitate fertilization, it may also limit the effective use of frozen–thawed semen by causing premature capacitation (65).

Short-term storage of buck sperm, at 5 °C in glucose-based extenders, resulted in faster rates of capacitation and acrosome reactions after 48–72 h, accompanied by increased lipid peroxidation and decreased membrane integrity. However, the addition of fructose to the extender was beneficial in reducing above mentioned effect (66). It was also shown that adding arginine to the extender enhanced capacitation and acrosome reactions and upregulated motility and capacitation-related genes (67). Similarly, pyridoxine supplementation has been reported to enhance motility, promote *in vitro* capacitation, and elevate acrosome reactions during cryopreservation (68).

A study examining the proteomic profiles of ram and buck sperm identified more than 2,100 proteins, of which 238 were differentially expressed between the two species (69). Among these, a higher abundance of capacitation-inhibitory proteins, such as SERPINE2, a serine protease inhibitor known to delay the capacitation cascade, was detected in goat sperm (69). In contrast, ram sperm showed lower SERPINE2 expression despite a generally high abundance of capacitation-regulatory proteins, suggesting the presence of species-specific regulatory mechanisms (69). Furthermore, the relatively rapid capacitation observed in buck sperm indicates that certain inhibitory regulatory pathways may be downregulated or modulated by biochemical and signaling stimuli.

### 3.4 Boar (pig)

In boar, sperm capacitation generally follows the conserved mammalian signaling cascade involving bicarbonate and calcium influx, activation of the sAC–cAMP/PKA pathway, and subsequent hyperactivation and acrosomal priming (70). However, boar sperm differ from other farm animal species by their marked sensitivity to *in vitro* conditions, due to a plasma membrane enriched in PUFA and low cholesterol content, which accelerated capacitation (70, 71). Despite this tendency, premature capacitation is physiologically restrained *in vivo* through zinc signature–dependent mechanisms that regulate sperm attachment to the oviduct and control the timing of acrosomal remodeling (72). In addition, seminal plasma components such as fibronectin-1 (FN1) and post-transcriptional regulation by sperm-derived microRNAs contribute to membrane stabilization, redox balance, and resistance to cryo- and *in vitro*–induced capacitation, collectively defining a boar-specific capacitation profile (73–75).

The PUFA-rich, highly fluid plasma membrane predisposes boar sperm to rapid *in vitro* capacitation, necessitating tight physiological regulation *in vivo*. Zinc signatures reflect distinct Zn^2+^ distribution patterns associated with functional capacitation states; early signatures (Sig-1/2) stabilize membranes and suppress premature capacitation, whereas later signatures (Sig-3/4) permit membrane remodeling and acrosomal priming near ovulation. Proteomic profiles indicated a zinc-centered regulatory network, with dynamic modulation of zinc-interacting proteins involved in motility, metabolism, and signaling, accordingly distinguishing boar capacitation from that of other species (72, 76, 77).

Recent studies have identified FN1, Izumo sperm–egg fusion protein 1 (IZUMO1), and acrosin-binding protein (ACRBP) as key proteins involved in the regulation of boar sperm capacitation (78–80). High-fertility boars exhibited elevated FN1 levels in seminal plasma and spermatozoa, associating FN1 to sperm durability and fertilizing capacity under stress (73, 75, 81). IZUMO1 is localized in the sperm head and has been associated with capacitation-related processes, contributing to the preparation of sperm for the acrosome reaction (78). In addition, ACRBP is localized in the acrosomal region of the sperm head and has been reported to contribute to acrosomal stability during capacitation through the ubiquitin–proteasome system (79).

Also, small non-coding RNAs, particularly microRNAs (miRNAs), have emerged as regulatory molecules involved in sperm capacitation and may contribute to species-dependent differences in capacitation-associated signaling pathways (67, 82). Several miRNAs, miR-21-5p, miR-15a, miR-34c, and miR-185 have been directly associated with capacitation potential and membrane stability in boar semen. Notably, miR-21-5p targets vinculin, a cytoskeletal linker involved in sperm–oocyte interaction, thereby suppressing premature capacitation and stabilizing the plasma membrane under *in vitro* conditions (82). Importantly, boar sperm with high cryotolerance exhibited miRNA profiles that limit oxidative stress and spontaneous capacitation through PI3K–Akt and mTOR signaling, potentially distinguishing miRNA signatures as functional markers of cryocapacitation and capacitation control (80, 83, 84).

## 4 The impact of cryopreservation on capacitation

Cryopreservation induces a capacitation-like state in spermatozoa, marked by enhanced membrane fluidity and lipid peroxidation (85), subsequently linked to excessive reactive oxygen species (ROS) production and aberrant intracellular signaling (65). Changes occurring during cryopreservation may disrupt the normal regulation and timing of sperm capacitation, leading to premature capacitation-like changes known as cryocapacitation (65, 85). The degree of cryocapacitation varies among species. Bull and ram sperm are not very sensitive (42, 52), but buck sperm are somewhat sensitive (64). Boar sperm, on the other hand, are very sensitive to cryocapacitation (71) because their plasma membranes are high in polyunsaturated fatty acids and low in cholesterol (86) (Table 2).

**Table 2:** Comparative overview of sperm capacitation mechanisms across farm animal species.

Species	Approximate capacitation time	Dominant trigger(s)	Key molecular and biochemical features	References
Bull (*Bos taurus*)	3–6 h	Bicarbonate (HCO₃^−^) sensitivity and heparin-like glycosaminoglycans	Tyrosine phosphorylation and CatSper-mediated Ca^2+^ influx	(44, 45)
Ram (*Ovis aries*)	4–6 h	Cholesterol efflux and protein tyrosine phosphorylation	High susceptibility to oxidative stress; seminal plasma proteins	(46, 53, 69)
Buck (*Capra hircus*)	1–4 h (early changes within about 1 h)	HCO₃^−^/Ca^2+^-dependent activation of the sAC–cAMP–PKA pathway	Rapid and highly sensitive capacitation	(64, 69)
Boar (*Sus domesticus*)	1–2 h (rapid)	Strong bicarbonate influx, cholesterol efflux, and Ca^2+^ entry	Highly fluid and fragile membrane	(70, 72, 81)

At the molecular level, cryopreservation causes sudden changes in temperature and osmotic pressure, which cause sperm plasma membrane phase transitions that mess up the organization of lipids (87, 88). Changes in the sperm plasma membrane cause cholesterol efflux, lipid raft reorganization, and increased membrane fluidity. These changes are similar to the early stages of physiological capacitation and start capacitation-related phosphorylation cascades (85). Nevertheless, in contrast to physiological capacitation, cryocapacitation occurs in an uncontrolled and heterogeneous way. Excessive ROS production after thawing, caused by challenges with mitochondria and the activation of NADPH oxidase, decreases antioxidant defenses and accelerates lipid peroxidation, which makes the membrane even less stable (89–91). Because of this, oxidative imbalance changes signaling related to capacitation toward premature acrosomal destabilization and stops the energy-dependent control of capacitation by lowering the mitochondrial membrane potential and ATP availability (92, 93).

Sperm plasma membrane instability and abnormal calcium signaling also change where and how key proteins involved in capacitation, such as AKAP3, CABYR, and CatSper, work. These alterations lead to hyperactivation and acrosome exocytosis; however, they disrupt the temporal coordination of the capacitation cascade, resulting in fertilization failure (71, 85). To prevent cryocapacitation, it’s necessary to use methods that keep the membrane stable and the redox balance. To prevent premature capacitation of sperm before freezing and to improve post-thaw function, optimization of cooling and warming rates, careful selection of cryoprotectants, and the addition of antioxidants are recommended. Recent proteomic and lipidomic investigations underscore biomarkers such as fibronectin 1 (FN1), osteopontin (OPN), and glutathione peroxidases 4 and 5 (GPX4/5) as prospective targets for enhancing cryopreservation protocols designed to preserve fertilization competence (80, 86).

## 5 Conclusions and future directions

Sperm capacitation is a highly regulated biochemical and biophysical process that facilitates fertilization via membrane remodeling, bicarbonate and calcium influx, activation of the sAC–cAMP–PKA pathway, and subsequent protein phosphorylation. The core signaling cascade is preserved across farm animals; however, significant interspecies variations exist in regulatory mechanisms, timing, and susceptibility to oxidative stress and cryopreservation. To improve artificial insemination, cryopreservation, and *in vitro* fertilization protocols, it is important to know what regulatory features are unique to each species. Future research should focus on using integrative methods to track the progress of capacitation from early membrane remodeling to the ability to fertilize, as well as combining real-time imaging with *omic*-based analyses. Nevertheless, this review has several limitations. The available literature on sperm capacitation is not equally represented among livestock species. In addition, differences in experimental designs, capacitation protocols, and analytical methodologies across studies may limit direct comparisons between species. Therefore, further studies using standardized approaches are needed to clarify species-specific capacitation mechanisms.

## References

[1] Harrison RA Gadella BM. Bicarbonate-induced membrane processing in sperm capacitation. Theriogenology. (2005) 63:342–51. doi: 10.1016/j.theriogenology.2004.09.016 15626403

[2] Puga Molina LC Luque GM Balestrini PA Marín-Briggiler CI Romarowski A Buffone MG. Molecular basis of human sperm capacitation. Front Cell Dev Biol. (2018) 6:72. doi: 10.3389/fcell.2018.00072, 30105226 PMC6078053

[3] Bailey JL. Factors regulating sperm capacitation. Syst Biol Reprod Med. (2010) 56:334–48. doi: 10.3109/19396368.2010.512377, 20849222

[4] Purdy PH Graham JK Azevedo HC. Evaluation of boar and bull sperm capacitation and the acrosome reaction using flow cytometry. Anim Reprod Sci. (2022) 246:106846. doi: 10.1016/j.anireprosci.2021.106846, 34563407

[5] Kerns K Zigo M Drobnis EZ Sutovsky M Sutovsky P. Zinc ion flux during mammalian sperm capacitation. Nat Commun. (2018) 9:2061. doi: 10.1038/s41467-018-04523-y, 29802294 PMC5970269

[6] Skowronek MF Pietroroia S Silvera D Ford M Cassina A Lecumberry F . Morphometric analysis of the sperm midpiece during capacitation. Tissue Cell. (2025) 95:102866. doi: 10.1016/j.tice.2025.102866, 40157222

[7] Cross NL. Reorganization of lipid rafts during capacitation of human sperm. Biol Reprod. (2004) 71:1367–73. doi: 10.1095/biolreprod.104.030502, 15215196

[8] Flesch FM Gadella BM. Dynamics of the mammalian sperm plasma membrane in the process of fertilization. Biochimica et Biophysica Acta. (2000) 1469:197–235. doi: 10.1016/s0304-4157(00)00018-6, 11063883

[9] Leahy T Gadella BM. New insights into the regulation of cholesterol efflux from the sperm membrane. Asian J Androl. (2015) 17:561–7. doi: 10.4103/1008-682X.153309, 25926609 PMC4492045

[10] Simons K Toomre D. Lipid rafts and signal transduction. Nat Rev Mol Cell Biol. (2000) 1:31–9. doi: 10.1038/35036052, 11413487

[11] Serafini S O’Flaherty C. Novel insights into the lipid signalling in human spermatozoa. Hum Reprod. (2025) 40:1440–51. doi: 10.1093/humrep/deaf085, 40409756 PMC12314153

[12] Buffone MG Wertheimer EV Visconti PE Krapf D. Central role of soluble adenylyl cyclase and cAMP in sperm physiology. Biochim Biophys Acta Mol basis Dis. (2014) 1842:2610–20. doi: 10.1016/j.bbadis.2014.07.013, 25066614 PMC4262597

[13] Chen Y Cann MJ Litvin TN Iourgenko V Sinclair ML Levin LR . Soluble adenylyl cyclase as an evolutionarily conserved bicarbonate sensor. Science. (2000) 289:625–8. doi: 10.1126/science.289.5479.625, 10915626

[14] Visconti PE Krapf D de la Vega-Beltrán JL Acevedo JJ Darszon A. Ion channels, phosphorylation and mammalian sperm capacitation. Asian J Androl. (2011) 13:395–405. doi: 10.1038/aja.2010.69, 21540868 PMC3739340

[15] Delgado-Bermúdez A Yeste M Bonet S Pinart E. A review on the role of bicarbonate and proton transporters during sperm capacitation in mammals. Int J Mol Sci. (2022) 23:6333. doi: 10.3390/ijms23116333, 35683013 PMC9180951

[16] Lishko PV Mannowetz N. CatSper: a unique calcium channel of the sperm flagellum. Curr Opin Physio. (2018) 2:109–13. doi: 10.1016/j.cophys.2018.02.004, 29707693 PMC5914511

[17] Chung J-J Shim S-H Everley RA Gygi SP Zhuang X Clapham DE. Structurally distinct Ca2+ signaling domains of sperm flagella orchestrate tyrosine phosphorylation and motility. Cell. (2014) 157:808–22. doi: 10.1016/j.cell.2014.02.056, 24813608 PMC4032590

[18] Strünker T Goodwin N Brenker C Kashikar ND Weyand I Seifert R . The CatSper channel mediates progesterone-induced Ca2+ influx in human sperm. Nature. (2011) 471:382–6. doi: 10.1038/nature09769, 21412338

[19] Publicover S Harper CV Barratt C. [Ca2+] i signalling in sperm—making the most of what you've got. Nat Cell Biol. (2007) 9:235–42. doi: 10.1038/ncb0307-235, 17330112

[20] Albrizio M Lacalandra GM Cinone M. The role of bicarbonate in the modulation of capacitation, spontaneous acrosome reaction and motility of equine fresh and frozen spermatozoa. Theriogenology. (2022) 187:112–8. doi: 10.1016/j.theriogenology.2022.04.032, 35561466

[21] Ferreira J Belliveau H Steegborn C Buck J Levin LR. Updating the mechanism of bicarbonate (HCO₃^−^) activation of soluble adenylyl cyclase (sAC). Int J Mol Sci. (2025) 26:6401. doi: 10.3390/ijms26136401, 40650178 PMC12250328

[22] Jaiswal BS Conti M. Calcium regulation of the soluble adenylyl cyclase expressed in mammalian spermatozoa. Proc Natl Acad Sci USA. (2003) 100:10676–81. doi: 10.1073/pnas.1831008100, 12958208 PMC196863

[23] Navarrete FA García-Vázquez FA Alvau A Escoffier J Krapf D Sánchez-Cárdenas C . Biphasic role of calcium in mouse sperm capacitation signaling pathways. J Cell Physiol. (2015) 230:1758–69. doi: 10.1002/jcp.24873, 25597298 PMC4752735

[24] Stival C Puga Molina L d C Paudel B Buffone MG Visconti PE Krapf D. "Sperm capacitation and acrosome reaction in mammalian sperm". In: Buffone MG, editor Sperm Acrosome Biogenesis and Function during Fertilization, Springer, Cham. (2016). p. 93–106. 10.1007/978-3-319-30567-7_5 27194351

[25] Ayoub S Rivera Sanchez NDR Fischoeder J Balbach M Levin LR Buck J . Cyclic AMP rescue of motility in sperm devoid of soluble adenylyl cyclase. Int J Mol Sci. (2025) 26:1489. doi: 10.3390/ijms26041489, 40003956 PMC11855772

[26] Esposito G Jaiswal BS Xie F Krajnc-Franken MA Robben TJ Strik AM . Mice deficient for soluble adenylyl cyclase are infertile because of a severe sperm-motility defect. Proc Natl Acad Sci. (2004) 101:2993–8. doi: 10.1073/pnas.0400050101, 14976244 PMC365733

[27] Litvin TN Kamenetsky M Zarifyan A Buck J Levin LR. Kinetic properties of “soluble” adenylyl cyclase: synergism between calcium and bicarbonate. J Biol Chem. (2003) 278:15922–6. doi: 10.1074/jbc.M212475200, 12609998

[28] Arcelay E Salicioni AM Wertheimer E Visconti PE. Identification of proteins undergoing tyrosine phosphorylation during mouse sperm capacitation. Int J Dev Biol. (2008) 52:463–72. doi: 10.1387/ijdb.072555ea, 18649259

[29] Etkovitz N Rubinstein S Daniel L Breitbart H. Role of PI3-kinase and PI4-kinase in actin polymerization during bovine sperm capacitation. Biol Reprod. (2007) 77:263–73. doi: 10.1095/biolreprod.106.056705 17494916

[30] Chatterjee M Nandi P Ghosh S Sen PC. Regulation of tyrosine kinase activity during capacitation in goat sperm. Mol Cell Biochem. (2010) 336:39–48. doi: 10.1007/s11010-009-0261-8, 19802524

[31] Ficarro S Chertihin O Westbrook VA White F Jayes F Kalab P . Phosphoproteome analysis of capacitated human sperm: evidence of tyrosine phosphorylation of a kinase-anchoring protein 3 and valosin-containing protein/p97 during capacitation. J Biol Chem. (2003) 278:11579–89. doi: 10.1074/jbc.m202325200, 12509440

[32] Rotfeld H Hillman P Ickowicz D Breitbart H. PKA and CaMKII mediate PI3K activation in bovine sperm by inhibition of the PKC/PP1 cascade. Reproduction. (2014) 147:347–56. doi: 10.1530/REP-13-0560, 24398875

[33] Qu X Han Y Chen X Lv Y Zhang Y Cao L . Inhibition of 26S proteasome enhances AKAP3-mediated cAMP–PKA signaling during boar sperm capacitation. Anim Reprod Sci. (2022) 247:107079. doi: 10.1016/j.anireprosci.2022.107079, 36209601

[34] González-Fernández L Macías-García B Loux SC Varner DD Hinrichs K. Focal adhesion kinases and calcium/calmodulin-dependent protein kinases regulate protein tyrosine phosphorylation in stallion sperm. Biol Reprod. (2013) 88:138–12. doi: 10.1095/biolreprod.112.107078, 23595906

[35] Mohanty G Sanchez-Cardenas C Paudel B Tourzani DA Salicioni AM Santi CM . Differential role of bovine serum albumin and HCO3− in the regulation of GSK3 alpha during mouse sperm capacitation. Mol Hum Reprod. (2024) 30:gaae007. doi: 10.1093/molehr/gaae007, 38341666 PMC10914453

[36] Syifa N Yang J-T Wu C-S Lin M-H Wu W-L Lai C-W . Phosphoproteomics and bioinformatics analyses reveal key roles of GSK-3 and AKAP4 in mouse sperm capacitation. Int J Mol Sci. (2020) 21:7283. doi: 10.3390/ijms21197283, 33023073 PMC7582274

[37] Sun P Wang Y Gao T Li K Zheng D Liu A . Hsp90 modulates human sperm capacitation via the Erk1/2 and p38 MAPK signaling pathways. Reprod Biol Endocrinol. (2021) 19:39. doi: 10.1186/s12958-021-00723-2, 33663544 PMC7931335

[38] Salgado-Lucio ML Ramírez-Ramírez D Jorge-Cruz CY Roa-Espitia AL Hernández-González EO. FAK regulates actin polymerization during sperm capacitation via the ERK2/GEF-H1/RhoA signaling pathway. J Cell Sci. (2020) 133:jcs239186. doi: 10.1242/jcs.239186, 32107290

[39] Schiavi-Ehrenhaus LJ Romarowski A Jabloñski M Krapf D Luque GM Buffone MG. The early molecular events leading to COFILIN phosphorylation during mouse sperm capacitation are essential for acrosomal exocytosis. J Biol Chem. (2022) 298:101988. doi: 10.1016/j.jbc.2022.101988, 35487245 PMC9142561

[40] Balbach M Rossetti T Ferreira J Ghanem L Ritagliati C Myers RW . On-demand male contraception via acute inhibition of soluble adenylyl cyclase. Nat Commun. (2023) 14:637. doi: 10.1038/s41467-023-36119-6, 36788210 PMC9929232

[41] Hess KC Jones BH Marquez B Chen Y Ord TS Kamenetsky M . The “soluble” adenylyl cyclase in sperm mediates multiple signaling events required for fertilization. Dev Cell. (2005) 9:249–59. doi: 10.1016/j.devcel.2005.06.007, 16054031 PMC3082461

[42] Chamberland A Fournier V Tardif S Sirard M Sullivan R Bailey J. The effect of heparin on motility parameters and protein phosphorylation during bovine sperm capacitation. Theriogenology. (2001) 55:823–35. doi: 10.1016/s0093-691x(01)00446-0, 11245268

[43] Parrish J Susko-Parrish J Winer M First N. Capacitation of bovine sperm by heparin. Biol Reprod. (1988) 38:1171–80. doi: 10.1095/biolreprod38.5.1171, 3408784

[44] Calvete JJ Raida M Sanz L Wempe F Scheit K-H Romero A . Localization and structural characterization of an oligosaccharide O-linked to bovine PDC-109 quantitation of the glycoprotein in seminal plasma and on the surface of ejaculated and capacitated spermatozoa. FEBS Lett. (1994) 350:203–6. doi: 10.1016/0014-5793(94)00768-3, 8070564

[45] Hung PH Suarez SS. Alterations to the bull sperm surface proteins that bind sperm to oviductal epithelium. Biol Reprod. (2012) 87:88. doi: 10.1095/biolreprod.112.099721, 22837481 PMC4434996

[46] Pini T de Graaf SP Druart X Tsikis G Labas V Teixeira-Gomes AP . Binder of sperm proteins 1 and 5 have contrasting effects on the capacitation of ram spermatozoa. Biol Reprod. (2018) 98:765–75. doi: 10.1093/biolre/ioy032, 29415221

[47] Mostek-Majewska A Majewska A Janta A Ciereszko A. New insights into posttranslational modifications of proteins during bull sperm capacitation. Cell Commun Signal. (2023) 21:72. doi: 10.1186/s12964-023-01080-w, 37046330 PMC10091539

[48] Handarini R Baharun A Rahmi A Sudrajat D Anggraeni A Nurcholis N . Correlation of sperm motility, acrosome integrity, protamine deficiency, and DNA fragmentation in proven and unproven Friesian Holstein bulls. J Adv Vet Anim Res. (2024) 11:796–802. doi: 10.5455/javar.2024.k831, 39605780 PMC11590584

[49] Pardede BP Kusumawati A Pangestu M Purwantara B. Bovine sperm HSP-70 molecules: a potential cryo-tolerance marker associated with semen quality and fertility rate. Front Vet Sci. (2023) 10:1167594. doi: 10.3389/fvets.2023.1167594, 37621869 PMC10445158

[50] Pardede BP Setyawan EMN Said S Kusumawati A Purwantara B Pangestu M . A-kinase anchor protein 4 (proAKAP4): protein molecule–based fertility marker of Indonesian dairy bull and its correlation with frozen-thawed sperm quality. Vet Med Int. (2025) 2025:8367714. doi: 10.1155/vmi/8367714, 40041135 PMC11876519

[51] Kaya A Dogan S Vargovic P Kutchy NA Ross P Topper E . Sperm proteins ODF2 and PAWP as markers of fertility in breeding bulls. Cell Tissue Res. (2022) 387:159–71. doi: 10.1007/s00441-021-03529-1, 34762184

[52] Barrios B Fernández-Juan M Muiño-Blanco T Cebrián-Pérez JA. Immunocytochemical localization and biochemical characterization of two seminal plasma proteins that protect ram spermatozoa against cold shock. J Androl. (2005) 26:539–49. doi: 10.2164/jandrol.04172, 15955894

[53] Luna C Colás C Casao A Serrano E Domingo J Pérez-Pé R . Ram seminal plasma proteins contribute to sperm capacitation and modulate sperm–zona pellucida interaction. Theriogenology. (2015) 83:670–8. doi: 10.1016/j.theriogenology.2014.10.030, 25515364

[54] Chen Y-L Li C-Y Wang P-H Wang R Zhuo X Zhang Y . Comparative proteomic identification of ram sperm before and after İn vitro capacitation. Animals. (2024) 14:2363. doi: 10.3390/ani14162363, 39199899 PMC11350773

[55] Zalazar L Iniesta-Cuerda M Sánchez-Ajofrín I Garde JJ Valls AJS Cesari A. Recombinant SPINK3 improves ram sperm quality and *in vitro* fertility after cryopreservation. Theriogenology. (2020) 144:45–55. doi: 10.1016/j.theriogenology.2019.12.019, 31911322

[56] Luna C Mendoza N Casao A Pérez-Pé R Cebrián-Pérez JA Muiño-Blanco T. C-Jun N-terminal kinase and p38 mitogen-activated protein kinase pathways link capacitation with apoptosis and seminal plasma proteins protect sperm by interfering with both routes. Biol Reprod. (2017) 96:800–15. doi: 10.1093/biolre/iox017, 28379343

[57] Aitken RJ. Reactive oxygen species as mediators of sperm capacitation and pathological damage. Mol Reprod Dev. (2017) 84:1039–52. doi: 10.1002/mrd.22871, 28749007

[58] Miguel-Jiménez S Pina-Beltrán B Gimeno-Martos S Carvajal-Serna M Casao A Pérez-Pe R. NADPH oxidase 5 and melatonin: involvement in ram sperm capacitation. Front Cell Dev Biol. (2021) 9:655794. doi: 10.3389/fcell.2021.655794, 34026754 PMC8138477

[59] Aitken RJ Drevet JR. The importance of oxidative stress in determining the functionality of mammalian spermatozoa: a two-edged sword. Antioxidants. (2020) 9:111. doi: 10.3390/antiox9020111, 32012712 PMC7070991

[60] Peris-Frau P Martín-Maestro A Iniesta-Cuerda M Sánchez-Ajofrín I Cesari A Garde JJ . Cryopreservation of ram sperm alters the dynamic changes associated with *in vitro* capacitation. Theriogenology. (2020) 145:100–8. doi: 10.1016/j.theriogenology.2020.01.046, 32007635

[61] O’Flaherty C Scarlata E. Oxidative stress and reproductive function: the protection of mammalian spermatozoa against oxidative stress. Reproduction. (2022) 164:F67–78. doi: 10.1530/rep-22-0200, 37021966

[62] Ritar A. Seasonal changes in LH, androgens and testes in the male angora goat. Theriogenology. (1991) 36:959–72. doi: 10.1016/0093-691x(91)90321-4

[63] Somanath P Gandhi K. Role of calcium and calcium channels in progesterone induced acrosome reaction in caprine spermatozoa. Asian Australas J Anim Sci. (2002) 15:949–56. doi: 10.5713/ajas.2002.949

[64] Kouamo J Kharche S. A comparative study of parthenogenetic activation and *in vitro* fertilization of *in vitro* matured caprine oocytes. Iranian J Vet Res. (2015) 16:20–4. PMC4789234 27175145

[65] Kritaniya D Yadav S Swain DK Reddy AV Dhariya R Yadav B . Freezing-thawing induces deprotamination, cryocapacitation-associated changes; DNA fragmentation; and reduced progesterone sensitivity in buck spermatozoa. Anim Reprod Sci. (2020) 223:106628. doi: 10.1016/j.anireprosci.2020.106628, 33128908

[66] Gangwar C Kharche SD Mishra AK Saraswat S Kumar N Sikarwar AK. Effect of diluent sugars on capacitation status and acrosome reaction of spermatozoa in buck semen at refrigerated temperature. Trop Anim Health Prod. (2020) 52:3409–15. doi: 10.1007/s11250-020-02374-8, 32918161

[67] Sahoo B Gupta MK. Effect of arginine-induced motility and capacitation on RNA population in goat spermatozoa. Vet Res Commun. (2023) 47:1427–44. doi: 10.1007/s11259-023-10092-3, 37162640

[68] Daramola J Adekunle E Oke O Onagbesan O Iyasere O Williams T . Effects of pyridoxine supplementation or in combination with other antioxidants on motility, *in vitro* capacitation and acrosome reaction of goat buck spermatozoa during cryopreservation. Small Rumin Res. (2015) 131:113–7. doi: 10.1016/j.smallrumres.2015.08.007

[69] Zhu W Cheng X Ren C Chen J Zhang Y Chen Y . Proteomic characterization and comparison of ram (*Ovis aries*) and buck (*Capra hircus*) spermatozoa proteome using a data independent acquisition mass spectometry (DIA-MS) approach. PLoS One. (2020) 15:e0228656. doi: 10.1371/journal.pone.0228656, 32053710 PMC7018057

[70] Lacalle E Consuegra C Martínez CA Hidalgo M Dorado J Martínez-Pastor F . Bicarbonate-triggered *in vitro* capacitation of boar spermatozoa conveys an increased relative abundance of the canonical transient receptor potential cation (TRPC) channels 3, 4, 6 and 7 and of CatSper-γ subunit mRNA transcripts. Animals. (2022) 12:1012. doi: 10.3390/ani12081012, 35454259 PMC9031844

[71] Watson PF. The causes of reduced fertility with cryopreserved semen. Anim Reprod Sci. (2000) 60:481–92. doi: 10.1016/s0378-4320(00)00099-3, 10844218

[72] Keller A Kerns K. Sperm capacitation as a predictor of boar fertility. Mol Reprod Dev. (2023) 90:594–600. doi: 10.1002/mrd.23690, 37306038

[73] Fraser L Wasilewska-Sakowska K Zasiadczyk Ł Piątkowska E Karpiesiuk K. Fractionated seminal plasma of boar ejaculates analyzed by LC–MS/MS: its effects on post-thaw semen quality. Genes. (2021) 12:1574. doi: 10.3390/genes12101574, 34680969 PMC8536186

[74] Salas-Huetos A Maghsoumi-Norouzabad L James ER Carrell DT Aston KI Jenkins TG . Male adiposity, sperm parameters and reproductive hormones: an updated systematic review and collaborative meta-analysis. Obes Rev. (2021) 22:e13082. doi: 10.1111/obr.13082, 32705766

[75] Vilagran I Yeste M Sancho S Castillo J Oliva R Bonet S. Comparative analysis of boar seminal plasma proteome from different freezability ejaculates and identification of fibronectin 1 as sperm freezability marker. Andrology. (2015) 3:345–56. doi: 10.1111/andr.12009, 25678437

[76] Machado SA Sharif M Kadirvel G Bovin N Miller DJ. Adhesion to oviduct glycans regulates porcine sperm Ca2+ influx and viability. PLoS One. (2020) 15:e0237666. doi: 10.1371/journal.pone.0237666, 32822385 PMC7442259

[77] Zigo M Kerns K Sen S . Zinc is a master-regulator of sperm function associated with binding, motility, and metabolic modulation during porcine sperm capacitation. Commun Biol. (2022) 5:538. doi: 10.1038/s42003-022-03485-8, 35660793 PMC9166710

[78] Hernández-Falcó M Sáez-Espinosa P López-Botella A Robles-Gómez L García-Vázquez F Izquierdo-Rico M . Immunolocalization and proteomic analyses of IZUMO1 in porcine spermatozoa. Front Cell Dev Biol. (2025) 13:1576881. doi: 10.3389/fcell.2025.1576881, 40469420 PMC12134388

[79] Zigo M Netherton J Zelenkova N Kerns K Kraus V Postlerová P . Bottom-up approach to deciphering the targets of the ubiquitin-proteasome system in porcine sperm capacitation. Sci Rep. (2024) 14:20159. doi: 10.1038/s41598-024-71056-4, 39215164 PMC11364869

[80] Zhao Y Wang Y Guo F Lu B Sun J Wang J . iTRAQ-based proteomic analysis of sperm reveals candidate proteins that affect the quality of spermatozoa from boars on plateaus. Proteome Sci. (2021) 19:9. doi: 10.1186/s12953-021-00177-9, 34330296 PMC8323236

[81] Rungruangsak J Suwimonteerabutr J Buranaamnuay K Asawakarn S Chantavisoote N Pisitkun T . 52 Difference of seminal plasma and sperm proteins in good and poor freezability boar ejaculates. Vet Stanica. (2022) 53:113–26. doi: 10.1071/rdv30n1ab52

[82] Xie Y Xu Z Wu C Zhou C Zhang X Gu T . Extracellular vesicle-encapsulated miR-21-5p in seminal plasma prevents sperm capacitation via vinculin inhibition. Theriogenology. (2022) 193:103–13. doi: 10.1016/j.theriogenology.2022.09.014, 36156422

[83] Deng C-Y Lv M Luo B-H Zhao S-Z Mo Z-C Xie Y-J. The role of the PI3K/AKT/mTOR signalling pathway in male reproduction. Curr Mol Med. (2021) 21:539–48. doi: 10.2174/1566524020666201203164910, 33272176

[84] Tang X Chen Y Luo H Bian Q Weng B Yang A . miR-126 controls the apoptosis and proliferation of immature porcine sertoli cells by targeting the pik3r2 gene through the PI3K/AKT signaling pathway. Animals. (2021) 11:2260. doi: 10.3390/ani11082260, 34438716 PMC8388524

[85] Benko F Ďuračka M Baňas Š Lukáč N Tvrdá E. Biological relevance of free radicals in the process of physiological capacitation and cryocapacitation. Oxygen. (2022) 2:164–76. doi: 10.3390/oxygen2020014

[86] Yeste M. Sperm cryopreservation update: Cryodamage, markers, and factors affecting the sperm freezability in pigs. Theriogenology. (2016) 85:47–64. doi: 10.1016/j.theriogenology.2015.09.047, 26506124

[87] Saha A Asaduzzaman M Bari FY. Cryopreservation techniques for ram sperm. Vet Med Int. (2022) 2022:7378379. doi: 10.1155/2022/7378379, 35535035 PMC9078814

[88] Xu B Wang R Wang Z Liu H Wang Z Zhang W . Evaluation of lipidomic change in goat sperm after cryopreservation. Front Vet Sci. (2022) 9:1004683. doi: 10.3389/fvets.2022.1004683, 36337197 PMC9630556

[89] Irigoyen P Mansilla S Castro L Cassina A Sapiro R. Mitochondrial function and reactive oxygen species production during human sperm capacitation: unraveling key players. FASEB J. (2024) 38:e23486. doi: 10.1096/fj.202301957rr, 38407497

[90] Luna C Serrano E Domingo J Casao A Pérez-Pé R Cebrián-Pérez J . Expression, cellular localization, and involvement of the pentose phosphate pathway enzymes in the regulation of ram sperm capacitation. Theriogenology. (2016) 86:704–14. doi: 10.1016/j.theriogenology.2016.02.024, 27063053

[91] Shi H Li Q-Y Li H Wang H-Y Fan C-X Dong Q-Y . ROS-induced oxidative stress is a major contributor to sperm cryoinjury. Hum Reprod. (2024) 39:310–25. doi: 10.1093/humrep/dead250 38011909

[92] Kadirvel G Kathiravan P Kumar S. Protein tyrosine phosphorylation and zona binding ability of *in vitro* capacitated and cryopreserved buffalo spermatozoa. Theriogenology. (2011) 75:1630–9. doi: 10.1016/j.theriogenology.2011.01.003, 21458055

[93] Kadirvel G Kumar S Kumaresan A. Lipid peroxidation, mitochondrial membrane potential and DNA integrity of spermatozoa in relation to intracellular reactive oxygen species in liquid and frozen-thawed buffalo semen. Anim Reprod Sci. (2009) 114:125–34. doi: 10.1016/j.anireprosci.2008.10.002, 19010614

[94] Cormier N Bailey JL. A differential mechanism is involved during heparin-and cryopreservation-induced capacitation of bovine spermatozoa. Biol Reprod. (2003) 69:177–85. doi: 10.1095/biolreprod.102.011056, 12620931

[95] de Lamirande E Gagnon C. Impact of reactive oxygen species on spermatozoa: a balancing act between beneficial and detrimental effects. Hum Reprod. (1995) 10:15–21. doi: 10.1093/humrep/10.suppl_1.15, 8592032

[96] Visconti PE Bailey JL Moore GD Pan D Olds-Clarke P Kopf GS. Capacitation of mouse spermatozoa: I. Correlation between the capacitation state and protein tyrosine phosphorylation. Development. (1995) 121:1129–37. doi: 10.1242/dev.121.4.1129, 7743926

